# Nanoprobe‐Enabled Precision Imaging and Panoramic Boundary Delineation in Retinoblastoma

**DOI:** 10.1002/cai2.70083

**Published:** 2026-10-11

**Authors:** Xuefei He, Precious Amarachi Ikwuagwu, Wenjing Pang, Aomen Chen, Lingchao Xiang, Feng Chen, Chen Xu, Aiguo Wu

**Affiliations:** ^1^ Department of Ophthalmology, Ningbo No.2 Hospital Wenzhou Medical University Ningbo China; ^2^ Ningbo Key Laboratory of Biomedical Imaging Probe Materials and Technology, Zhejiang International Cooperation Base of Biomedical Materials Technology and Application, Zhejiang Engineering Research Center for Biomedical Materials at Ningbo Cixi Institute of Biomedical Engineering Laboratory of Advanced Theranostic Materials and Technology, Ningbo Institute of Materials Technology and Engineering, Chinese Academy of Sciences Ningbo China; ^3^ University of Chinese Academy of Sciences Beijing China

**Keywords:** multimodal imaging, nanoprobes, precision diagnosis, retinoblastoma, structural and functional boundary

## Abstract

Retinoblastoma is the most common primary intraocular malignancy in children. Its prognosis depends critically on accurate delineation of the tumor and its margins, early detection of small lesions, and timely monitoring of therapeutic response. Traditional single‐modality imaging techniques are inherently constrained by trade‐offs among spatial resolution, molecular sensitivity, and dynamic imaging capability, making them insufficient for the demands of precision diagnosis and treatment. In recent years, nanomedicine‐based imaging strategies have emerged as promising approaches to overcome these limitations. Through rational design of nanoprobes with tunable physicochemical properties, active targeting capabilities, and integrated multimodal signaling, researchers have achieved more comprehensive visualization and diagnostic evaluation of retinoblastoma lesions. In this review, we systematically summarize the major imaging modalities applied to retinoblastoma, including optical imaging, ultrasound (US), magnetic resonance imaging (MRI), and photoacoustic imaging (PAI). Subsequently, the corresponding imaging probes and targeting strategies were discussed, which were developed to enhance the imaging contrast and improve the spatiotemporal resolution of tumor visualization. Furthermore, we highlight the emerging role of artificial intelligence (AI) in the rational design of high‐performance imaging nanoprobes and in multimodal image analysis, emphasizing its potential to enable quantitative tumor and boundary delineation. Finally, we outline the key challenges that must be addressed to advance the clinical translation of nanoprobes, including the identification of novel targeting strategies, AI‐assisted image analysis, the development of models that more accurately recapitulate the human ocular microenvironment, and the application of new biocompatible materials. Overall, this review aims to provide comprehensive and insightful guidance for the development of next‐generation nanoprobes for the precise visualization and boundary delineation of retinoblastoma.

## Graphical abstract

This review highlights that nanoprobe‐enabled multimodal imaging, combined with targeted probe design and AI‐assisted analysis, significantly enhances the precise visualization and boundary delineation of retinoblastoma. It further outlines key challenges and directions for translating high‐performance imaging nanoprobes into clinical practice. (The graphical abstract image was generated by Procreate.)

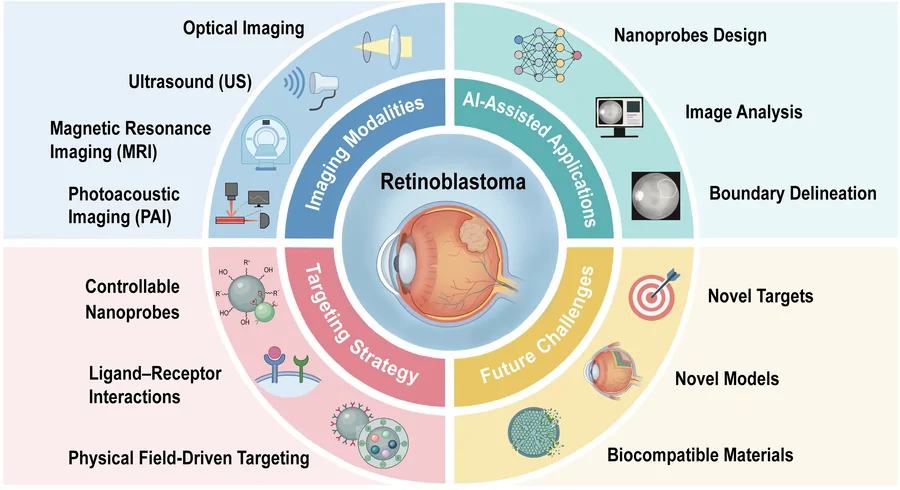

**Abbreviations**
AI: artificial intelligence
BRB: blood–retinal barrier
DOX: doxorubicin
EPR: enhanced permeability and retention
FLI: fluorescence imaging
ICG: indocyanine green
LSPR: localized surface plasmon resonance
MRI: magnetic resonance imaging
NIR: near‐infrared
NIR‐II: near‐infrared‐II
OCT: optical coherence tomography
PAI: photoacoustic imaging
PFP: perfluoropentane
QD: quantum dot
TA: tannic acid
US: ultrasonography

## 1 Introduction

Retinoblastoma is a rare, yet highly aggressive intraocular malignancy of childhood, accounting for approximately 3.0%–6.1% of all pediatric cancers [1, 2, 3]. Over the past decades, its clinical management has evolved from enucleation‐centered radical treatment toward globe‐sparing strategies that aim to maximize visual preservation while maintaining stringent oncologic control [4, 5, 6]. The success of this transition depends largely on the accurate, reproducible, and quantitative assessment of tumor burden and spatial extent [7, 8]. Therapeutic decision‐making, including ophthalmic artery or intravitreal chemotherapy, laser photocoagulation, cryotherapy, pars plana vitrectomy (PPV), and radiotherapy, requires precise delineation of tumor size, location, and, critically, high‐risk features such as infiltrative margins and vitreous or subretinal seeding [9, 10, 11]. In parallel, long‐term prognostic management relies on dynamic imaging surveillance to detect minimal residual disease, early recurrence, or progressive dissemination at the earliest possible stage. Thus, improvements in overall survival and visual preservation are closely linked to imaging‐based early diagnosis, accurate staging, and reliable treatment monitoring.

Boundaries in Biomedicine, especially in the field of tumor boundary research, represent a newly proposed research direction in recent years. In the context of retinoblastoma, tumor boundary delineation extends beyond simple visualization of tumor contours [12, 13, 14]. Instead, it represents a multidimensional and dynamic characterization of tumor extent, biological activity, molecular heterogeneity, and therapeutic accessibility [15, 16]. This expanded understanding is especially important because retinoblastoma often shows complex growth patterns, including endophytic or exophytic expansion, subretinal fluid, vitreous seeding, retinal infiltration, optic nerve involvement, and treatment‐induced regression, all of which may obscure the true tumor margin on conventional structural imaging. From this perspective, retinoblastoma boundaries can be classified into several interrelated dimensions. Morphological boundaries describe the anatomical extent of visible tumor tissue and its spatial relationship with adjacent ocular structures. These boundaries are essential for tumor staging, volume assessment, and local treatment planning. However, morphological information alone may be insufficient because the apparent structural margin does not always correspond to the biologically active tumor front. Functional boundaries reflect spatial changes in tumor‐associated physiological processes, such as oxygenation status, metabolic activity, and hypoxia. These parameters may identify aggressive tumor regions beyond the clearly visible anatomical margin. Molecular boundaries are defined by the spatial distribution of tumor‐specific biomarkers, receptor expression, or enzymatic activity, thereby providing information on cellular heterogeneity and potential therapeutic targets. In addition, therapeutic boundaries describe the regions effectively reached by drugs, nanoprobes, photothermal or photodynamic activation, or other therapeutic interventions. This concept is particularly relevant for evaluating whether a treatment adequately covers the entire viable tumor region while sparing normal retinal tissue. Clinically, accurate tumor boundary delineation is not merely an imaging objective but a critical prerequisite for precision management of retinoblastoma. Precise visualization of tumor extent facilitates early diagnosis, accurate disease staging, individualized treatment planning, image‐guided intervention, and longitudinal assessment of therapeutic response [17]. More importantly, reliable boundary delineation helps distinguish viable tumor tissue from treatment‐induced regression or scar formation, thereby reducing unnecessary interventions while minimizing damage to normal retinal structures [18]. Ultimately, improved boundary characterization contributes toward higher eye‐preservation rates, better visual outcomes, and more personalized clinical management [19]. Therefore, an ideal imaging strategy should integrate multiple imaging modalities and multifunctional nanoprobes to achieve comprehensive characterization of retinoblastoma boundaries rather than relying solely on structural imaging.

Currently, the imaging evaluation of retinoblastoma primarily integrates clinical examination with multimodal techniques, including optical imaging (e.g., indirect ophthalmoscopy, RetCam, and optical coherence tomography (OCT)), ultrasonography (US), and magnetic resonance imaging (MRI), with increasing incorporation of functional and molecular modalities such as photoacoustic imaging (PAI) and fluorescence imaging (FLI) [20, 21]. Although computed tomography (CT) is sensitive for detecting calcification, it is no longer recommended in international guidelines due to the associated ionizing radiation risks [22]. Each modality offers distinct advantages in terms of spatial and temporal resolution, imaging sensitivity, and penetration depth [23]. OCT provides micrometer‐scale axial resolution, enabling layer‐resolved retinal assessment and detection of subtle lesions; however, its limited penetration restricts visualization of deeper intraocular structures and anterior segment involvement. US offers real‐time imaging with moderate tissue penetration and can identify characteristic features such as calcification; yet, it is constrained by limited soft tissue contrast and operator‐dependent boundary interpretation [24]. MRI, owing to its superior soft tissue contrast, remains essential for comprehensive intraocular evaluation and detection of high‐risk features [25, 26, 27]; however, its sensitivity to early molecular alterations and microscopic lesions remains limited [28, 29]. FLI shows strong potential for molecularly specific imaging, but is affected by tissue scattering and absorption, as well as photobleaching and phototoxicity [30]. PAI, a hybrid modality combining optical absorption contrast with ultrasonic detection, enables functional assessment of vascular architecture and oxygenation through hemoglobin‐related signals, potentially achieving a balance between spatial resolution and imaging depth [31]. However, quantitative stability and boundary accuracy may be compromised not only by interference from photoacoustic signals generated by weak laser absorption in subretinal fluid components (proteins, lipids, or hemoglobin) but also by a fundamental limitation: the imaging depth is largely confined to superficial regions due to scattering of focused illumination and tissue‐induced attenuation of high‐frequency ultrasound components. Traditional examinations can identify endophytic tumors, but often fail to detect the subtle grayish masses associated with early exophytic growth [24]. The rare diffuse infiltrating subtype is particularly susceptible to misdiagnosis as uveitis [32, 33]. Moreover, the biologically active region of the tumor often extends beyond the clinically visible morphological margin, thereby defining a functionally relevant boundary. This functional interface, characterized by aberrant proliferation, altered metabolism, and active remodeling of the tumor microenvironment, typically surpasses the structural boundary delineated by conventional imaging modalities [34]. Therefore, single‐modality imaging is generally insufficient to comprehensively delineate both structural and functional tumor extents or to simultaneously achieve deep penetration, high resolution, and molecular specificity.

Nanotechnology‐enabled imaging probes have emerged as a promising strategy to improve imaging contrast and provide panoramic characterization of retinoblastoma lesions and their boundaries [35]. Through rational modulation of nanoprobe size, morphology, surface charge, hydrophilicity, and surface functionalization, it is possible to improve in vivo stability, amplify imaging signals, and reduce background interference, thereby enhancing detection sensitivity for small lesions and improving the reproducibility of boundary delineation [36, 37, 38]. Moreover, multifunctional nanoprobes that integrate magnetic, optical, and acoustic components within a single platform also allow complementary multimodal imaging [39]. In such systems, structural imaging modalities provide precise anatomical localization, whereas functional or molecular modalities offer insights into perfusion status, hypoxia, metabolic activity, and target expression. This combined approach establishes a feasible framework for the comprehensive delineation of both structural and functional tumor boundaries [12, 40].

However, effective visualization of retinoblastoma and its margins using nanoprobes critically depends on achieving sufficient and specific intratumoral accumulation. Passive targeting strategies relying solely on the enhanced permeability and retention (EPR) effect often result in limited and heterogeneous probe enrichment within intraocular tumors [41]. Consequently, efforts to enhance boundary imaging performance have shifted from passive accumulation toward active targeting strategies. These include ligand receptor‐mediated recognition to promote selective uptake and retention, endogenous stimulus‐responsive systems triggered by pH, redox conditions, or enzymatic activity, and exogenous physical field‐driven approaches utilizing magnetic, optical, or acoustic stimuli [42, 43, 44, 45]. These strategies aim to improve detection sensitivity at infiltrative margins and micro‐disseminated foci while enhancing consistency and reliability in boundary delineation.

Simultaneously, advances in artificial intelligence (AI) are also influencing nanoprobe design and multimodal imaging analysis. AI‐assisted materials' discovery combines high‐throughput experimentation with multiscale computational modeling to generate multidimensional data sets that permit virtual screening and multi‐objective optimization [46, 47, 48]. This strategy contributes to the development of predictive “structure–property and in vivo behavior” models, thereby facilitating the identification of high‐contrast probes tailored for intraocular delivery [49]. In nanoprobe‐assisted multimodal imaging, AI further supports cross‐modality registration, feature alignment, and integrative analysis, automated lesion segmentation, quantitative boundary assessment, and longitudinal evaluation of therapeutic response [50]. In addition, radiomics and deep learning techniques connect imaging phenotypes with underlying biological characteristics, improving prediction of tumor aggressiveness and recurrence risk [51, 52].

In this context, the present review summarizes recent advances in nanotechnology‐enabled precision imaging and comprehensive boundary delineation of retinoblastoma (Figure 1). We outline the principal imaging modalities used in retinoblastoma and discuss the contrast‐enhancement mechanisms of nanoprobes in US, MRI, PAI, and FLI, with particular emphasis on their contributions to small lesion detection and boundary accuracy. We then examine targeting strategies used to guide nanoprobe delivery and discuss the role of AI in probe design and multimodal imaging analysis. Finally, we address key translational challenges and propose future perspectives, aiming to establish a conceptual and methodological framework for the development of next‐generation nanoprobes capable of comprehensively characterizing both structural and functional boundaries in retinoblastoma.

**Figure 1:**
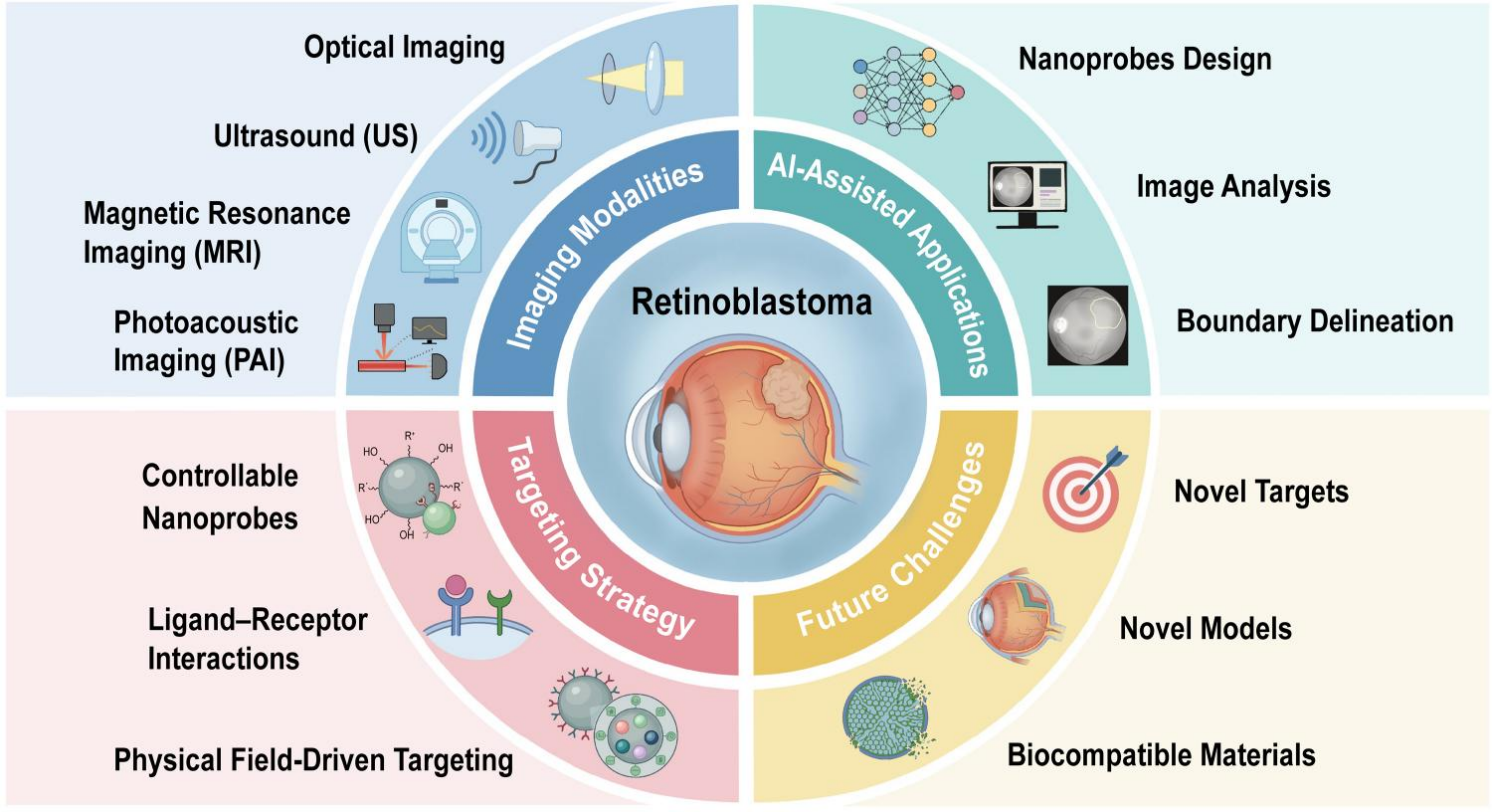
Imaging techniques and probe design strategies for retinoblastoma and its boundary identification. This figure was generated by Procreate.

## 2 Retinoblastoma‐Related Imaging Technologies

The diagnosis and treatment of retinoblastoma rely heavily on imaging technologies for accurate delineation of tumor boundaries, early identification of small lesions, and dynamic monitoring throughout the treatment process [53]. As illustrated in Figure 2 and Table 1, current imaging modalities primarily include optical imaging, US imaging, PAI, and MRI [20, 75]. Although these imaging technologies have broad applications across oncology, retinoblastoma presents several unique challenges that distinguish its boundary delineation from that of other solid tumors. First, the compact anatomical structure of the eye requires submillimeter imaging accuracy to preserve adjacent retinal tissues and visual function [68]. Second, the presence of the blood–retinal barrier substantially limits nanoprobe delivery and necessitates specialized targeting strategies [76]. Third, retinoblastoma frequently shows vitreous seeding, multifocal lesions, and treatment‐induced regression patterns, making tumor boundaries highly dynamic and heterogeneous rather than simple anatomical contours [77]. Furthermore, as a pediatric malignancy, imaging strategies must minimize invasiveness, radiation exposure, and systemic toxicity while maintaining high sensitivity and specificity [78]. These unique pathological and clinical characteristics collectively demand imaging systems capable of simultaneously resolving morphological, functional, and molecular boundaries with exceptional precision. However, traditional single imaging techniques are generally constrained by limitations such as insufficient penetration depth, weak imaging contrast, and ambiguous tumor boundary definition, posing significant challenges to the detection of small lesions and precise boundary determination [22]. To address these limitations, researchers have developed a series of nanoprobes to enhance the imaging contrast of tumor lesions across different technologies. Through surface functional modification, these nanoprobes can achieve targeted enrichment in tumor regions, amplify lesion signal intensity, and provide complementary information for distinguishing retinoblastoma lesions and defining tumor structural and functional boundaries, thereby significantly improving the accuracy and reliability of retinoblastoma imaging.

**Figure 2:**
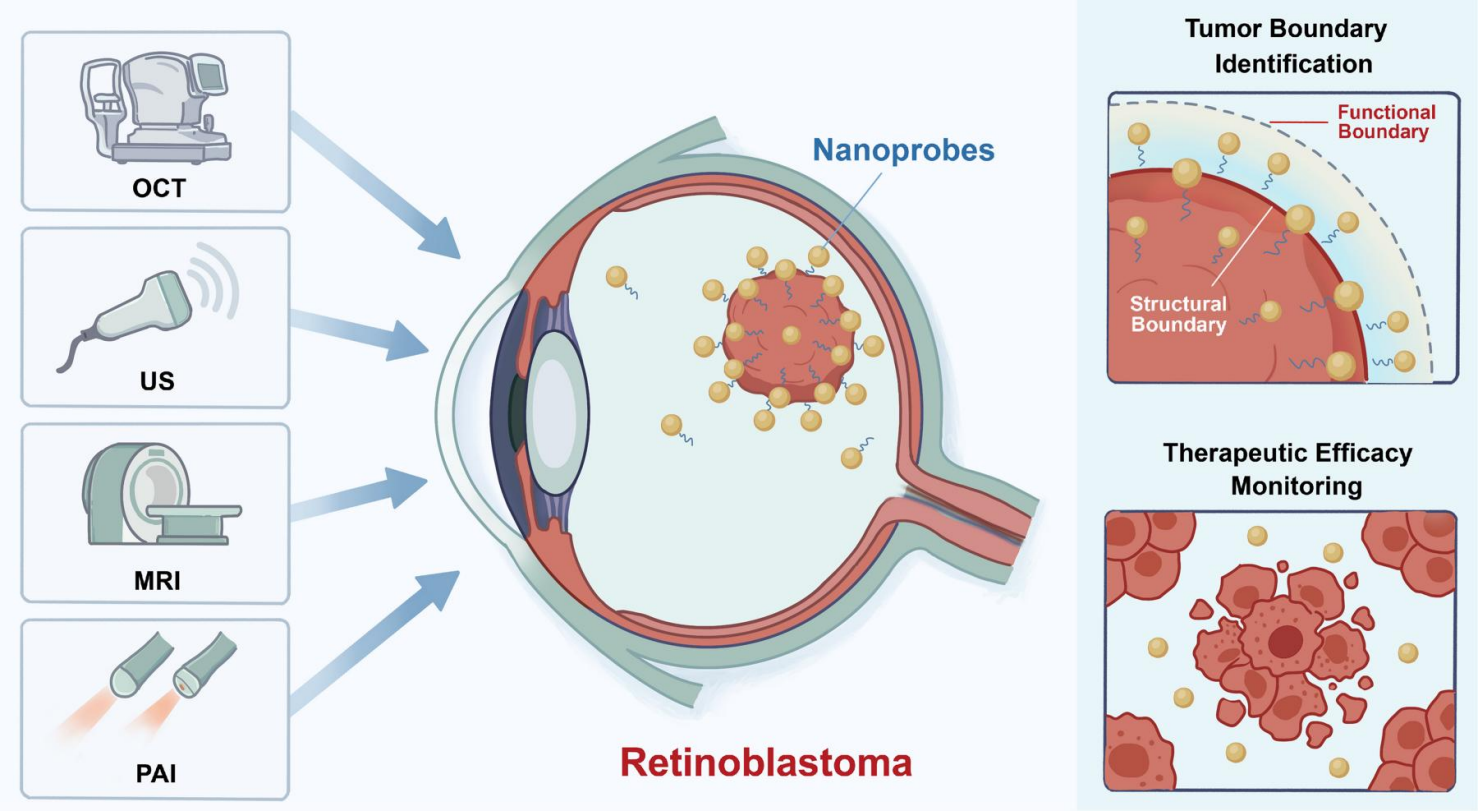
Schematic illustration of the imaging modalities for delineating the boundaries of retinoblastoma. Optical imaging, US, PA, and MRI provide complementary imaging capabilities, enhancing retinoblastoma boundary delineation. Functional imaging nanoprobes integrate diagnostic and therapeutic strategies, enabling precise identification of tumor boundaries and real‐time visualization of therapeutic efficacy, thereby supporting precision medicine for retinoblastoma. This figure was generated by Procreate. MRI, magnetic resonance imaging; OCT, optical coherence tomography; PAI, photoacoustic imaging; US, ultrasonography.

**Table 1:** Imaging modalities and their nanoprobes for retinoblastoma boundary delineation.

Imaging modality		Basic principle	Key advantages	Major limitations	Representative nano‐enhanced strategies	Key improvement for retinoblastoma	References
Optical Imaging	OCT	Low‐coherence interferometry detects backscattered light to reconstruct micrometer‐resolution retinal structure	Ultra‐high resolution; non‐invasive; real‐time imaging; and ideal for early microstructural detection and treatment monitoring	Limited penetration depth; relies on intrinsic scattering contrast; lacks molecular specificity; and difficulty distinguishing subtle tumor margins	Gold nanorods (GNRs), ligand‐modified GNRs, photothermal OCT probes, carbon nanodot clusters (CGNPs), RGD‐functionalized probes, and dye‐conjugated targeted probes	Enhanced backscattering and molecular targeting; improved lesion contrast; and enables precise micro‐boundary delineation	[54, 55, 56, 57, 58, 59]
	FLI	Fluorophore emission enables molecular‐level visualization of biomarkers and tumor cells	High sensitivity; molecular specificity; and useful for early diagnosis and treatment monitoring	Limited penetration depth; signal attenuation and scattering; and difficulty detecting vitreous seeds	AuNP‐based MEF probes, EpCAM–AuNP–siRNA systems, nitrogen‐doped carbon dots (N‐CDs), PLGA/PCL carriers, and ICG‐liposomes	Amplified fluorescence signals; targeted tumor recognition; and improved detection of vitreous disseminated and small retinoblastoma lesions	[36, 60, 61, 62, 63, 64, 65]
US		Acoustic reflection from tissue interfaces; contrast enhanced by acoustic impedance differences	Real‐time, non‐ionizing, deep penetration, convenient clinical use, and good soft tissue contrast	Low sensitivity to small lesions; limited microvascular visualization; and unclear tumor–normal boundary contrast	Phase‐change nanodroplets (PFP/PFH core) with lipid/polymer/protein shells; magnetic nanoparticle‐coupled phase‐change probes	Improved echo signal, enhanced tumor boundary delineation, and better detection of small retinoblastoma lesions and microenvironment features	[23, 66, 67]
MRI		Proton relaxation (T_1_/T_2_) differences generate high‐resolution soft tissue contrast and multiparametric functional imaging	Excellent soft tissue resolution; deep penetration; provides structural and functional information; and essential for staging and optic nerve invasion assessment	Long scan time; sedation required in children; limited BRB penetration of conventional contrast agents; and low sensitivity for small lesions	SPIONs (T_2_ shortening), dextran‐coated iron oxide nanoparticles (DCIONs), and functionalized BRB‐penetrating nanoprobes	Enhanced contrast and tumor targeting; improved detection of small/vitreous lesions; and supports imaging‐guided therapy	[17, 68, 69, 70, 71, 72]
PAI		Optical absorption converted into acoustic signal via thermoelastic expansion	Combines high optical contrast with ultrasound penetration; suitable for deep lesion imaging and functional assessment	Requires strong optical absorbers; limited endogenous contrast; and depends on exogenous agents for specificity	Gold nanoparticles (AuNPs), nanorods/nanostars, ligand‐targeted AuNPs (FA, HA), and BRB‐penetrating nanoprobes	Strong PA signal amplification; improved tumor localization and functional boundary mapping; and early micro‐lesion detection	[36, 55, 56, 63, 73, 74]

Abbreviations: AuNP, Au nanoparticles; BRB, blood–retinal barrier; FA, folate; FLI, fluorescence imaging; HA, hyaluronic Acid; ICG, indocyanine green; MRI, magnetic resonance imaging; OCT, optical coherence tomography; PAI, photoacoustic imaging; PFH, perfluorohexane; PFP, perfluoropropane; PLGA/PCL, Poly(lactic‐co‐glycolic acid)/Polycaprolactone; RGD, Arginine–Glycine–Aspartic acid; SPIONs, Superparamagnetic iron oxide nanoparticles; US, ultrasonography.

### 2.1 Optical Imaging

In the clinical assessment of retinoblastoma, indirect ophthalmoscopy and RetCam imaging remain fundamental methods for initial diagnosis. Advanced optical techniques, most notably optical coherence tomography (OCT) and fluorescence imaging (FLI), are emerging as pivotal tools [79]. These techniques overcome traditional optical limitations by allowing microstructural and molecular visualization to support theranostics applications. OCT demonstrates high sensitivity in assessing the spatial relationship between the tumor and adjacent retinal structures, including the macula, detecting subretinal fluid, and distinguishing post‐therapeutic scarring from tumor recurrence, thereby supporting clinical decision‐making and longitudinal follow‐up [80]. FLI relies on the administration of fluorescent agents for direct visualization of tumor vascular perfusion patterns and leakage characteristics [81, 82]. It provides valuable information regarding tumor vascular distribution and growth features, offering insights into the hemodynamic activity and structural integrity of tumor‐associated vessels [83, 84]. By delineating these vascular characteristics, FLI can be used to evaluate therapeutic response and guide treatment strategy selection. OCT and FLI are frequently integrated with complementary imaging modalities to achieve a more comprehensive delineation of tumor margins and biological characteristics, thereby enhancing diagnostic accuracy and the reliability of therapeutic monitoring [85].

#### 2.1.1 Optical Coherence Tomography

Optical Coherence Tomography (OCT) is a non‐contact, non‐ionizing, high‐resolution imaging modality that enables real‐time and repeatable in vivo visualization of intraocular structures, thereby avoiding the tissue damage associated with invasive procedures such as biopsy [86]. Owing to these advantages, OCT has become an important tool for monitoring disease progression and evaluating therapeutic response in retinoblastoma [87].

OCT is particularly valuable for detecting retinoblastoma growth patterns that are difficult to recognize by conventional fundus examination. Certain subtypes, especially exophytic retinoblastoma growing into the subretinal space and diffuse infiltrating retinoblastoma spreading in a plaque‐like manner within the retinal layers without forming a discrete mass, may remain confined to the inner or outer retina and therefore fail to produce obvious fundus elevation [78]. The absence of a well‐defined tumor mass makes these lesions particularly challenging to identify ophthalmoscopically [5, 32]. OCT can identify early tumor lesions and delineate their boundaries by revealing focal retinal thickening and microarchitectural disorganization, particularly within the ganglion cell layer (GCL) and the inner plexiform layer (IPL) [88]. Based on three‐dimensional volumetric scanning, both en face reconstructions and B‐scan images typically demonstrate localized retinal thickening in retinoblastoma, and thickness gradients can serve as quantitative indicators for tumor boundary delineation [89]. Building on this capability, automated retinal thickness quantification and segmentation algorithms have been integrated with animal‐positioning systems, enabling high‐throughput, quantitative, and dynamic monitoring of tumor growth in retinoblastoma mouse models [54]. During treatment, OCT can further assess restoration of retinal lamination and normalization of retinal thickness, thereby helping to determine whether complete tumor regression has been achieved [90]. Its high repeatability and stability facilitate a transition from qualitative observation to objective, quantitative assessment of tumor and its boundaries [91, 92].

In addition, the introduction of exogenous nanoprobes to enhance OCT contrast has attracted increasing attention [93]. Gold nanorods (GNRs) have been widely investigated because their localized surface plasmon resonance (LSPR) can enhance backscattered signals from target regions, thereby improving OCT imaging contrast in pathological tissues [73]. Early studies reported that intravitreally injected GNRs largely remained confined within the vitreous cavity, demonstrating limited penetration across the inner limiting membrane into the retinal parenchyma; these findings also raised concerns about potential intraocular inflammatory responses that could compromise image interpretation. Subsequent studies have focused on optimizing nanoparticle size and surface functionalization to improve tissue permeability and biocompatibility [94]. Surface engineering strategies, including PEGylation, targeting ligand conjugation, and stimuli‐responsive design, have been shown to significantly improve circulation stability, tumor accumulation, and cellular uptake [95]. These optimized platforms have also been extended to photothermal OCT (PT‐OCT) applications. For instance, ligand‐functionalized GNRs can selectively bind pathological sites such as choroidal neovascularization and generate localized photothermal effects under laser excitation, thereby enhancing OCT signal intensity and enabling high‐contrast visualization of lesions [55].

#### 2.1.2 Fluorescence Imaging

Fluorescence imaging (FLI) is a noninvasive technique that uses fluorescent agents to visualize tumor‐associated biomarkers at the cellular and molecular levels [96]. In retinoblastoma, its clinical utility is illustrated by the intrinsic fluorescence of topotecan under ultraviolet light, which enables real‐time monitoring of drug distribution during periocular or intravitreal injections, as well as facilitating detection of inadvertent conjunctival exposure. This approach is practical, low cost, and easily implementable in clinical settings [97]. These features make FLI a valuable adjunct for therapeutic monitoring and procedural guidance in retinoblastoma management. Unlike structural imaging modalities, FLI primarily detects molecular abnormalities, allowing more precise discrimination between tumor tissue and normal retina through biomarker‐targeted probes [98, 99]. For example, nitrogen‐doped carbon dots (N‐CDs) have been developed as novel fluorescent probes that respond to metabolic abnormalities in retinoblastoma cells, such as elevated NAD^+^ levels, resulting in enhanced fluorescence and tumor‐specific recognition [60].

Recent advances in signal amplification have further expanded the sensitivity of FLI for detecting small tumor foci. Among these, fluorescence–chemiluminescence dual‐modal signal amplification technology combines fluorescence and chemiluminescence enhancement to achieve cascaded signal amplification, thereby overcoming the limitations of conventional fluorescent probes, such as weak signals and high background noise. This strategy improves the detection sensitivity of low‐abundance biomarkers, including MDM2, E2F3, or mutant Retinoblastoma1 proteins, making it particularly suitable for monitoring vitreous‐disseminated tumor cells and small residual lesions within the vitreous cavity [61, 62]. Although FLI provides excellent molecular sensitivity for identifying tumor‐associated biomarkers, its limited tissue penetration restricts its ability to independently delineate deep tumor margins [13]. Therefore, fluorescence imaging is more suitable as a molecular complement to structural imaging modalities such as MRI or OCT, particularly in image‐guided surgery and intraoperative margin assessment.

### 2.2 Ultrasound Imaging

Ultrasound (US) imaging is a non‐invasive modality characterized by real‐time visualization, non‐ionizing radiation, ease of operation, and favorable soft tissue contrast. Because of its relatively deep tissue penetration and high spatial resolution, US has become a routine tool for the diagnosis of retinoblastoma [75, 100]. Conventional US can display the echogenic features and blood flow signals of retinoblastoma lesions, providing important clinical value for initial tumor screening and qualitative assessment. However, several limitations remain. In particular, US has limited sensitivity for diffuse infiltrating retinoblastoma and may fail to detect small lesions or accurately delineate low‐contrast tumor boundaries [101, 102]. On the one hand, its sensitivity to lesions with subtle echogenic differences is inherently constrained by the spatial resolution limits imposed by the acoustic diffraction barrier, making it difficult to distinguish subtle structural disparities between tumors and surrounding normal tissues, particularly for minimally elevated or plaque‐like lesions. On the other hand, its ability to depict intratumoral micro‐blood flow and the tumor microenvironment is limited by the physical resolution limit of conventional ultrasound (approximately 200 μm), which prevents reliable detection of smaller microvessels.

To address these limitations, contrast‐enhanced ultrasound technology has emerged. Traditional ultrasound contrast agents are typically gas‐filled microbubbles with a shell–core structure that enhance acoustic impedance mismatch and thereby improve delineation of tumor boundaries and internal architecture [103]. Although clinically mature agents such as lipid‐shelled microbubbles are widely used, their inherently short intravascular circulation time, susceptibility to rupture, and limited ability to penetrate vessel walls for tissue extravasation restrict their effectiveness in complex tumor microenvironments [104, 105]. As an alternative, phase‐change nanodroplet‐based contrast agents have been proposed [106]. These nanodroplets typically consist of a liquid perfluorinated compound core (e.g., perfluoropropane [PFP], perfluorohexane [PFH]) stabilized by lipid, polymer, or protein shells [106, 107, 108, 109, 110]. For instance, nanodroplets composed of a perfluorobutane (PFB) core and a phospholipid shell containing DSPC and DSPE‐PEG_2000_, with Pluronic F68 added to control surface tension can be efficiently prepared by spontaneous nucleation and show good storage stability [107]. These nanodroplets undergo a controllable liquid‐to‐gas phase transition under low‐intensity ultrasound, forming highly echogenic microbubbles that significantly enhance local acoustic signals. Compared with traditional microbubbles, phase‐change nanodroplets offer longer circulation time, improved tissue extravasation, and stronger contrast enhancement, thereby showing promise for the detection of small retinoblastoma lesions and more accurate delineation of tumor boundaries [66].

### 2.3 Magnetic Resonance Imaging

MRI, as a noninvasive imaging modality that provides deep tissue penetration, exceptional soft tissue contrast, and non‐ionizing radiation, is indispensable for the diagnosis and management of pediatric retinoblastoma [68]. It supplies multi‐parameter structural and functional information that is valuable for initial tumor diagnosis, staging assessment, evaluation of local invasion, and follow‐up monitoring of treatment efficacy [17]. In conventional MRI sequences, retinoblastoma typically shows intermediate to relatively high signal intensity on T_1_‐weighted images, low signal intensity on T_2_‐weighted images, and significant enhancement on contrast‐enhanced scans. These sequences clearly depict the anatomical relationships between the tumor and adjacent structures, including the sclera, choroid, and vitreous cavity, thereby facilitating evaluation of tumor boundaries, vascularity, and invasive spread [69, 70].

However, MRI faces certain clinical limitations in retinoblastoma management. These include relatively long scan times, the frequent need for sedation or anesthesia in pediatric patients, and contraindications in some patients with metallic implants. In addition, conventional gadolinium‐based contrast agents show limited tumor‐specific accumulation and insufficient tissue penetration because of the blood–retinal barrier (BRB) and the occult nature of small retinoblastoma lesions [111]. These constraints reduce their sensitivity for early detection and accurate assessment of small lesions [112]. Among various nanoprobe systems, iron‐based nanoprobes have been studied most extensively. In particular, superparamagnetic iron oxide nanoprobes (SPIONs) can significantly shorten the T_2_ relaxation time and enhance T_2_‐weighted imaging contrast, thereby improving visualization of retinoblastoma and facilitating more precise delineation of tumor boundaries [71]. These findings suggest that MRI primarily contributes to anatomical boundary definition but remains insufficient for identifying microscopic tumor infiltration, emphasizing the necessity of integrating molecular imaging modalities.

### 2.4 Photoacoustic Imaging

PAI is an innovative non‐invasive imaging modality that combines the high contrast of optical imaging with deep tissue penetration of ultrasound [113]. By exploiting the optical absorption properties of biological tissues, PAI converts absorbed light energy into acoustic signals for image formation [114]. Owing to its intrinsic ability to generate high‐contrast images at depths beyond the reach of conventional optical techniques, PAI has been increasingly explored across a range of challenging anatomical sites, such as the brain, where accurate visualization of small or deeply located lesions is essential [115, 116]. These characteristics are highly relevant for retinoblastoma detection, where early identification of small intraocular lesions and clear delineation of tumor boundaries are critical for treatment planning and vision preservation.

To enhance PAI contrast and sensitivity, various exogenous nanoprobes have been developed. These nanoprobes primarily improve acoustic signal generation by increasing near‐infrared light absorption, while their targeting efficiency and biocompatibility can be regulated through particle size, morphology, and surface functionalization. Among available candidates, gold nanoparticles (AuNPs) are among the most widely studied because of their tunable local surface plasmon resonance (LSPR), strong optical absorption, and favorable scattering properties [55, 56, 74]. Upon laser excitation, their LSPR property enables efficient conversion of light into heat and subsequently into acoustic waves, thereby generating strong PAI signals that meet the imaging needs of deep retinal tissues. By tailoring the size and morphology of AuNPs, local PAI signals can be further enhanced, improving the precision of tumor boundary delineation. For example, Kim et al. [73] confirmed that intravenously administered 20 nm AuNPs could successfully penetrate the BRB, whereas 100 nm nanoprobes were undetectable in retinal tissue. The smaller nanoparticles showed stable association with retinal neuron cell membranes (75% ± 5%), endothelial cells (17% ± 6%), and periendothelial glial cells (8% ± 3%). This selective cellular distribution allows PAI to differentiate retinoblastoma tissue from normal retinal tissue and define functional tumor boundaries with greater accuracy. Additionally, surface functionalization of AuNPs with retinoblastoma‐targeting ligands, such as folic acid or hyaluronic acid, can further enhance tumor recognition, thereby improving imaging specificity and sensitivity. Such strategies provide robust support for the early detection of small retinoblastoma lesions [36, 63]. These observations collectively indicate that photoacoustic imaging bridges the gap between purely structural imaging and molecular imaging by simultaneously providing anatomical and functional boundary information. Clinically, this capability may facilitate early identification of hypoxic tumor regions, evaluation of treatment response, and individualized therapeutic planning [117].

### 2.5 Multimodal Imaging

Different imaging modalities have distinct advantages and limitations in terms of spatial resolution, tissue penetration depth, and functional specificity. For instance, US imaging and MRI primarily provide structural information, such as tumor location, size, and anatomical extent, whereas PAI and FLI reveal functional or molecular information related to tissue metabolism, oxygenation status, and biomarker expression [53]. Accordingly, multimodal imaging provides complementary structural and functional information, enabling more comprehensive characterization of retinoblastoma and its microenvironment.

Recent advances in nanoprobe engineering have greatly accelerated the development of multimodal imaging platforms by integrating multiple imaging components and targeting ligands within a single system. Among these, liposome‐based systems have shown particular value because of their biocompatibility, payload flexibility, and ability to combine imaging and therapeutic functions. For example, the FA‐DOX‐ICG‐PFP@Lip nanoplatform, which combines indocyanine green (ICG) for PAI/FLI and perfluoropentane (PFP) for US imaging, enables PAI/US/FLI tri‐modal imaging. This platform allows simultaneous acquisition of spatial structural information and metabolic imaging signals, significantly improving the accuracy of tumor boundary delineation [118]. Another promising strategy involves liposome–hydrogel hybrid systems, which combine the reservoir function of natural polymer hydrogels with the penetration capability of liposomal nanocarriers to achieve imaging‐guided, on‐demand drug release, and precise coverage of the tumor. Lin et al. [119] developed a near‐infrared (NIR)‐responsive QD Lipo/ICG/LAgel hybrid platform, in which ICG, DOX, and quercetin were co‐encapsulated in a low‐gelation‐temperature agarose hydrogel to form a hydrogel–liposome composite nanoprobe. In this system, the photothermal effect of ICG triggers hydrogel depolymerization, leading to the precise release of drug‐loaded liposomes under 808 nm NIR irradiation. ICG serves as a dual‐modal fluorescence/photoacoustic imaging agent, enabling in vivo intraocular fluorescence imaging for tumor localization and, when combined with PAI, simultaneous visualization of structural and functional tumor boundaries. These liposomes, with a particle size of ~107 nm, can diffuse through the vitreous network, accumulate at the tumor boundary, and exert synergistic chemo‐photothermal effects. During treatment, real‐time fluorescence imaging further allows dynamic monitoring of drug distribution and tumor regression.

Polymer‐based nanocarriers have also emerged as versatile theranostic platforms for retinoblastoma. PLGA–PFP nanoparticles (FPTP), co‐loaded with ferric tannic acid (Fe^3+^TA) and paclitaxel (PTX), integrate US imaging, photothermal therapy, and PTX‐based chemotherapy. In vitro experiments confirmed that these nanoparticles can simultaneously enhance ultrasound signals, generate laser‐triggered hyperthermia, and induce synergistic antitumor effects against Y79 retinoblastoma cells [120]. Similarly, Huang et al. [121] developed Fe^3+^TA/PLGA/Ce6 nanoparticles that use chlorin e6 (Ce6) for photoacoustic imaging as well as photothermal/photodynamic therapy, whereas Fe^3+^TA provides an additional chemodynamic therapeutic effect. Under laser irradiation, this multifunctional nanoplatform achieved efficient ablation of Y79 cells, highlighting the potential of a PAI‐guided photothermal/chemodynamic strategy for integrated diagnosis and treatment of retinoblastoma.

In summary, multimodal imaging leverages the effective integration of structural and functional imaging to improve retinoblastoma detection, boundary delineation, and treatment monitoring. Multimodal imaging enables complementary characterization of different boundary dimensions, integrating morphological, functional, and molecular information to achieve comprehensive delineation of retinoblastoma. By reducing the limitations of any single modality, nanoprobe‐enabled multimodal platforms provide a more integrated framework for precision diagnosis and therapy. Continued integration of MRI, PAI, and NIR‐II fluorescence technologies, multimodal image‐guided precision diagnosis and treatment systems for retinoblastoma are expected to accelerate clinical translation, providing stronger technical support for eye‐preserving treatment and personalized therapeutic strategies.

### 2.6 Theranostic Nanoprobes for Imaging‐Guided Precision Therapy in Retinoblastoma

Beyond improving tumor visualization and panoramic boundary delineation, multifunctional nanoprobes are increasingly evolving into theranostic platforms that integrate multimodal imaging with therapeutic interventions within a single nanosystem [122]. In contrast to conventional contrast agents that mainly provide diagnostic information, theranostic nanoprobes not only provide high‐resolution anatomical and molecular information for accurate tumor localization but also enable image‐guided therapy, real‐time monitoring of therapeutic responses, and dynamic assessment of treatment efficacy. This integration is particularly valuable for retinoblastoma, where complete tumor eradication must be achieved while preserving the surrounding retinal structures and visual function [123]. Consequently, imaging‐guided theranostic nanoprobes have emerged as an important strategy for improving treatment precision and minimizing off‐target toxicity.

The incorporation of therapeutic agents into multimodal imaging nanoplatforms allows simultaneous diagnosis and treatment within a single nanosystem. Representative examples include liposomal nanoprobes co‐loading doxorubicin (DOX), indocyanine green (ICG), and perfluoropentane (PFP), which integrate FLI, PAI, and US imaging with chemotherapy and photothermal therapy [118]. In such systems, multimodal imaging allows real‐time visualization of nanoprobes' accumulation within tumor tissues, thereby enabling precise identification of tumor boundaries before treatment. Meanwhile, the phase‐transition property of PFP enhances ultrasound contrast and facilitates stimulus‐responsive drug release, which may improve local therapeutic efficacy while reducing systemic toxicity. Similarly, multifunctional liposomal platforms incorporating quantum dots, ICG, and therapeutic cargos have demonstrated the feasibility of combining FLI with near‐infrared‐triggered therapy [119]. These imaging‐guided therapeutic systems provide opportunities to accurately determine the optimal therapeutic window, monitor therapeutic activation, and evaluate treatment responses noninvasively, which is particularly advantageous for ophthalmic tumors requiring highly localized intervention. Another representative strategy involves metal‐polyphenol‐based theranostic nanoplatforms, such as Fe^3+^‐tannic acid‐containing nanoparticles loaded with photosensitizers [120]. These systems combine PAI with multiple therapeutic modalities, including photodynamic therapy (PDT), photothermal therapy (PTT), and chemodynamic therapy (CDT). Owing to their intrinsic imaging capability, nanoprobe distribution and retention within tumor tissues can be monitored throughout treatment, allowing dynamic evaluation of therapeutic progression and facilitating individualized treatment optimization. At the same time, the synergistic interactions among different therapeutic mechanisms further enhance antitumor efficacy while overcoming the limitations associated with single‐modality treatment. From the perspective of retinoblastoma management, such platforms may be useful for improving local tumor control while minimizing damage to adjacent normal ocular tissues.

Compared with conventional imaging agents that primarily provide diagnostic information, theranostic nanoprobes establish a closed‐loop strategy integrating diagnosis, therapeutic intervention, and treatment evaluation [124]. Before treatment, multimodal imaging can delineate tumor morphology, molecular distribution, and functional heterogeneity, thereby defining the spatial range requiring intervention. During treatment, imaging can guide drug release, photothermal activation, photodynamic irradiation, or other stimulus‐responsive processes with improved spatial precision. After treatment, repeated imaging can assess tumor regression, detect residual lesions, and identify early recurrence. Such an integrated workflow is expected to substantially improve treatment precision and support personalized management of retinoblastoma. Collectively, theranostic nanoprobes represent a natural extension of nanoprobe‐enabled precision imaging. By integrating multimodal imaging with chemotherapy, PTT, PDT, CDT, or other therapeutic strategies, these platforms provide a promising approach for imaging‐guided personalized treatment of retinoblastoma.

## 3 Strategies for Enhancing Retinoblastoma Boundary Imaging

When nanoprobes are applied to enhance delineation of retinoblastoma boundaries across different imaging modalities, efficient and selective tumor accumulation is a prerequisite for achieving high imaging contrast and accurate lesion identification. Current strategies for improving nanoprobe delivery to retinoblastoma can be broadly classified into three categories: passive targeting, active targeting, and multi‐physical‐field‐driven targeting. Passive targeting, which serves as the fundamental strategy, relies on the enhanced permeability and retention (EPR) effect to achieve non‐specific tumor accumulation. In contrast, active targeting improves selectivity through ligand‐mediated recognition of tumor‐associated biomarkers. Multi‐physical‐field‐driven targeting further enhances nanoprobe delivery by using external magnetic, optical, or acoustic stimuli to modulate tissue permeability or direct probe enrichment at the tumor site [36].

### 3.1 Passive Targeting Strategies

A passive targeting strategy primarily leverages the EPR effect, a pathological feature commonly observed in solid tumors. This phenomenon arises from abnormal tumor angiogenesis, which results in disorganized and highly permeable vasculature, together with impaired lymphatic drainage. By tailoring the physicochemical properties of nanoprobes, relatively tumor‐selective accumulation can be achieved without the need for specific molecular targeting ligands. This approach offers advantages such as a relatively simple preparation and favorable biocompatibility. Early research mainly focused on optimizing the physicochemical properties of nanoprobes to maximize EPR‐mediated tumor accumulation. In general, particle size is controlled within the range of 10–200 nm to facilitate vascular extravasation and intratumoral retention. Surface charge is often adjusted to a neutral or slightly negative state, and polyethylene glycol (PEG) modification is introduced to reduce plasma protein adsorption and clearance by the mononuclear phagocytic system (MPS). Collectively, these strategies prolong blood circulation time and increase the likelihood of passive tumor accumulation.

However, despite improving the relative concentration of nanoprobes within tumor tissue, passive targeting lacks specific tumor‐cell recognition and therefore provides limited selectivity and enrichment efficiency. This limitation is particularly relevant in retinoblastoma, where precise diagnosis and safety are critical. Accordingly, with the growing understanding of tumor biology and the identification of retinoblastoma‐associated biomarkers, research has progressively shifted toward more selective targeting strategies.

### 3.2 Active Targeting Strategies

Active targeting strategies rely on functionalization of nanoprobes with specific ligands that recognize and bind to receptors overexpressed on retinoblastoma cells. Through ligand–receptor interactions and subsequent receptor‐mediated endocytosis, these nanoprobes achieve enhanced cellular internalization, improved penetration across the BRB, and preferential accumulation within tumor tissue, while effectively minimizing non‐specific tissue distribution and potential off‐target toxicity [125]. Consequently, active targeting strategies have become the mainstream of current research, encompassing receptor‐mediated targeting, intelligent responsive strategies, and multi‐physical‐field‐driven targeting approaches.

Among these, receptor‐mediated targeting remains the most widely studied and technologically mature approach. It involves conjugating nanoprobes with targeting molecules, such as peptides, aptamers, antibodies, or small‐molecule ligands, allowing selective recognition of tumor‐related biomarkers expressed on retinoblastoma cells. Such specific interactions enhance intracellular uptake and imaging signals, facilitating precise delineation of tumor location, size, and biological characteristics [118, 126]. Compared with other malignancies, active targeting strategies for retinoblastoma should preferentially exploit disease‐associated biomarkers expressed within the ocular tumor microenvironment or across the blood–retinal barrier [127]. Representative targets include GD2, EpCAM, nucleolin, integrins, and angiogenesis‐associated molecules, which have demonstrated potential for improving nanoprobe accumulation, molecular boundary visualization, and image‐guided precision therapy [127, 128, 129, 130, 131]. Therefore, intelligent responsive nanoprobes have been developed by exploiting distinctive features of the tumor microenvironments in order to further improve targeting precision. Gao et al. [132] designed a receptor‐mediated targeted intelligent response nanoprobe, AMD‐GCCNPs‐DOX, based on the high expression of C–X–C chemokine receptor 4 (CXCR4) in retinoblastoma. Under acidic conditions, the nano‐ceria shows oxidase‐like activity and induces reactive oxygen species (ROS) generation, whereas high intracellular glutathione (GSH) levels cleave disulfide bonds and trigger controlled release of DOX. This design enables synergistic enhancement of both imaging and therapeutic efficacy. In vivo experiments showed that, following an intravitreal injection, the probe specifically accumulated at the tumor site in the Y79/GFP‐luc xenograft model, allowing clear tracking of tumor progression by bioluminescence imaging. In a p107s genetic model, it effectively inhibited tumor growth, reduced vascular leakage, and caused no obvious damage to normal retinal structure, demonstrating the precision advantages of combining receptor‐mediated targeting with a microenvironmental response mechanism. Zheng et al. [133] developed NIR‐II fluorescent nanoprobes (Combo‐NPs) based on a TPA‐BD polymer loaded with lenvatinib. This platform responds to the acidic tumor microenvironment and promotes vascular‐normalization therapy, while NIR‐II fluorescence imaging enables clear visualization of tumor vasculature and growth patterns. In a subcutaneous tumor model, maximal fluorescence intensity at the tumor site was observed at 24 h post‐administration, and the probes demonstrated sufficient tissue penetration for deep ocular tissue imaging. Li et al. [134] further designed reduction‐responsive polymer micelles loaded with melphalan, in which the core and shell were linked by disulfide bonds. In the glutathione (GSH)‐rich microenvironment of retinoblastoma, cleavage of these bonds triggers drug release and simultaneously increases fluorescence intensity, enabling tumor‐specific imaging and therapy. Collectively, an active targeting strategy improves not only nanoprobe accumulation but also the accuracy of molecular boundary identification. However, the heterogeneous expression of tumor biomarkers and dynamic changes in the tumor microenvironment remain important challenges that limit clinical translation.

### 3.3 Multi‐Physical‐Field‐Driven Targeting Strategies

In addition to biochemical targeting approaches, multi‐physical‐field‐driven targeting strategies utilize externally applied magnetic, optical, or acoustic fields to promote nanoprobe accumulation at tumor sites, thereby further improving tumor‐targeting efficiency. Compared with conventional targeting methods, physical‐field‐assisted strategies offer a means of overcoming the BRB through reversible and spatially controllable modulation. For example, magnetic nanoprobes can generate localized hyperthermic effects under an alternating magnetic field, leading to a temporary and reversible increase in BRB permeability. Studies have shown that intra‐ophthalmic arterial administration of transferrin‐modified magnetic nanoparticles, followed by application of an external alternating magnetic field, induces a localized thermal effect that enhances retinal penetration of injected fluorescent agents [135]. Importantly, this strategy improves intraocular delivery through physical modulation rather than permanent chemical disruption of the BRB, thereby offering potential advantages in controllability and biosafety [135].

## 4 Artificial Intelligence‐Assisted Multifunctional Nanoprobe Design and Multimodal Image Analysis

### 4.1 AI‐Enabled Efficient Development of Multifunctional Imaging Probes With High Performance

Multifunctional nanoprobes integrate multiple imaging modalities with targeting ligands and activatable imaging mechanisms within a single nanosystem, supporting more precise and personalized diagnosis and therapy. They represent a major direction in nanomedicine. However, the conventional development of these probes still relies heavily on empirical approaches and trial‐and‐error experimentation. Researchers must iteratively explore vast combinations of material compositions, structural parameters (e.g., size, morphology, and surface functionalization), imaging functionality, and in vivo behaviors, an approach that is both time‐consuming and costly. More importantly, such a paradigm often fails to establish generalizable design principles that quantitatively relate composition and structure to imaging performance and biological fate.

In recent years, AI‐driven materials' discovery has accelerated the convergence of materials science and biomedical imaging, establishing new paradigms for the rational development of multifunctional nanoprobes [136, 137]. AI is a broad computational framework encompassing machine learning (ML), deep learning (DL), reinforcement learning, and more recently, foundation and generative models [138]. ML algorithms identify complex relationships from structured data sets, whereas DL architectures, particularly convolutional neural networks (CNNs), graph neural networks (GNNs), and transformer‐based models, can automatically extract hierarchical features from multidimensional biomedical data [139]. By learning from high‐dimensional data sets, AI can transform conventional experience‐based screening into predictive, optimizable, and data‐guided workflows, thereby shortening the development cycles of new probes and facilitating the identification of high‐performance candidates [115]. Representative studies have demonstrated the practical value of AI in nanomedicine development. For example, Chan et al. [46] developed a transformer‐based neural network capable of predicting lipid nanoparticle formulations with improved nucleic acid delivery efficiency, significantly reducing experimental screening and accelerating formulation optimization. Likewise, Shan et al. [47] summarized AI‐enabled strategies integrating high‐throughput experimentation, active learning, and predictive modeling to establish closed‐loop workflows for nanomedicine discovery. More recently, Shen et al. [48] highlighted the application of machine learning throughout nanoparticle formulation design, characterization, and quality optimization, demonstrating the broad potential of AI across the entire nanoprobe development pipeline. Within this framework, AI‐guided probe design supports the selection of highly sensitive imaging materials, improves contrast performance, and ultimately strengthens delineation of both structural and functional tumor boundaries.

Practically, multidimensional materials databases can be established through combinatorial chemistry and high‐throughput screening, while molecular docking and molecular dynamics simulations support rapid pre‐screening and prioritization of candidate probes. Recent AI‐assisted nanoprobe development generally follows a closed‐loop optimization workflow, involving high‐throughput data generation, feature engineering, predictive model construction, virtual screening, experimental validation, and iterative model updating [140]. Bayesian optimization, active learning, and graph neural networks have shown particular promise for efficient exploration of large chemical design spaces while minimizing experimental burden [141]. On this basis, machine learning models can then be trained to establish quantitative relationships among nanoprobe structure, physicochemical properties, and biological behavior. Parameters such as particle size, surface charge, surface functionalization, and ligand density can thus be linked to imaging sensitivity, targeting efficiency, and in vivo distribution. This provides a quantitative basis for multi‐objective optimization. These approaches are particularly relevant for retinoblastoma, where small lesion size and delicate ocular anatomy impose stringent requirements on probe specificity and signal‐to‐noise ratio. AI can rapidly identify tumor‐targeting ligands and optimize affinity and selectivity [142, 143], while also guiding the integration of endogenous microenvironment‐responsive triggers, such as enzyme state, pH, and redox state, with exogenous stimuli, including magnetic, optical, and acoustic fields [144]. Such rationally designed activatable probes may substantially improve lesion‐to‐background contrast and enable more sensitive visualization of retinoblastoma boundaries.

### 4.2 AI‐Facilitated Multimodal Image Fusion for Panoramic and Precise Delineation of Tumor Boundaries

Multimodal imaging combines the complementary strengths of different imaging technologies to simultaneously provide both anatomical structural and dynamic functional information, offering a key pathway for precision diagnosis and therapy. With the support of multifunctional nanoprobes, imaging modalities such as MRI, FLI, US, and PAI can be integrated within a single platform to enable coordinated acquisition of structural and molecular data. In principle, high‐spatial‐resolution structural imaging delineates morphological tumor margins and adjacent anatomy, whereas high‐sensitivity functional imaging reveals metabolic, hypoxic, or other microenvironmental features associated with tumor progression. Nevertheless, effective multimodal analysis remains challenging because different imaging modalities vary substantially in acquisition mechanism, spatial and temporal resolution, sensitivity, and noise characteristics. These discrepancies complicate cross‐modal registration, feature alignment, and quantitative integration, thereby limiting comprehensive assessment of tumors and their boundaries.

AI has increasingly been applied to oncologic image analysis to enable automated, quantitative, and reproducible tumor assessment. In multimodal image analysis, CNN‐based segmentation models, transformer‐based architectures, and multimodal fusion networks have demonstrated remarkable performance in lesion segmentation, image registration, super‐resolution reconstruction, and quantitative boundary extraction [145]. These approaches can effectively integrate complementary anatomical and molecular information from MRI, OCT, PAI, and fluorescence imaging, thereby improving tumor boundary delineation and treatment‐response assessment [146]. For example, deep learning‐based image registration frameworks, such as SuperCUT, have substantially improved cross‐modal image alignment while preserving fine structural details [50]. In retinoblastoma, machine learning methods have been used for automated screening and grading of fundus images, as well as fine segmentation and longitudinal quantification of lesions and key ocular structures on high‐resolution images, supporting efficient screening and treatment assessment [147]. Beyond this, radiomics and deep learning can extract high‐dimensional imaging features and associate them with molecular and cellular characteristics, offering predictive information on tumor aggressiveness, therapeutic response, and recurrence risk [148, 149]. Therefore, AI‐assisted ocular image analysis can improve both diagnostic accuracy and analytical efficiency. For nanoprobe‐driven multimodal imaging, in particular, AI‐based multi‐level fusion strategies offer additional advantages. By integrating information across different imaging levels, AI can improve cross‐modal registration, reduce noise, enhance feature extraction, and strengthen the robustness of boundary recognition [150]. As a result, AI‐assisted multimodal analysis can provide a more panoramic and precise delineation of retinoblastoma, including both its structural extent and functional heterogeneity. This capability is expected to further support image‐guided precision diagnosis, treatment planning, and therapeutic monitoring. Nevertheless, widespread clinical implementation remains limited by the scarcity of large‐scale annotated retinoblastoma data sets, insufficient external validation, limited model interpretability, and concerns regarding data standardization and privacy. Future studies should emphasize multicenter collaboration and explainable AI frameworks to facilitate clinical translation. Rather than replacing clinicians, AI is expected to transform tumor boundary delineation from qualitative visual interpretation to quantitative, data‐driven clinical decision support.

Beyond image interpretation and multimodal data fusion, AI is increasingly being integrated into the image acquisition and reconstruction pipeline to improve the intrinsic performance of current imaging systems [151]. DL‐based reconstruction algorithms can effectively suppress imaging noise, compensate for undersampled data, and recover fine anatomical details that may otherwise be lost during image acquisition, thereby improving image quality without requiring substantial hardware modifications [152]. In addition, AI‐powered super‐resolution techniques, particularly those based on CNNs and transformer architectures, have demonstrated remarkable capability in reconstructing high‐resolution images from low‐resolution inputs by learning complex spatial features from large‐scale data sets [153]. These approaches offer a promising strategy to overcome the spatial resolution limitations of existing imaging modalities while preserving structural fidelity. Furthermore, AI can facilitate intelligent optimization of imaging hardware through adaptive parameter selection, automated focusing, beamforming optimization, and real‐time signal enhancement [154]. Such intelligent acquisition strategies can improve signal‐to‐noise ratio, imaging depth, acquisition efficiency, and spatial resolution across multiple imaging modalities, including MRI, photoacoustic imaging, ultrasound, and optical imaging. Rather than replacing existing imaging equipment, AI is expected to complement current hardware by maximizing imaging performance through computational enhancement and data‐driven optimization [155]. For retinoblastoma imaging, these advances may substantially improve the visualization of tiny vitreous seeds, infiltrative tumor margins, and early recurrent lesions that are often difficult to identify using conventional imaging alone. Combined with AI‐assisted multifunctional nanoprobe design and multimodal image analysis, AI‐enabled image reconstruction and intelligent imaging systems are expected to further enhance precise tumor boundary delineation and facilitate earlier diagnosis, individualized treatment planning, and real‐time therapeutic monitoring.

## 5 Conclusion

Current imaging modalities remain insufficient for the early detection of retinoblastoma and the accurate delineation of tumor boundaries. Across commonly used diagnostic approaches, including optical imaging, US, MRI, and PAI, there is an urgent need for novel imaging probes that combine high spatial resolution, high specificity, and the capability to traverse BRB. These characteristics are essential for improving early diagnosis of retinoblastoma and achieving accurate delineation of both structural and functional boundaries of tumors. From the perspective of nanoprobe engineering, rational optimization of probe size, surface charge, ligand functionalization, and stimulus‐responsive behavior can substantially improve targeted delivery, BRB penetration, and intratumoral retention. In particular, the incorporation of targeting ligands, such as folate and transferrin ligands, together with exogenous stimulus‐responsive components, has shown considerable promise for promoting selective accumulation and local signal amplification. Furthermore, by integrating multimodal imaging, nanoprobes enable the transition from single‐modality imaging to multimodal imaging. This approach provides complementary anatomical, functional, and metabolic information at the molecular, cellular, and tissue levels, significantly improving early detection and prognostic visualization of retinoblastoma. Similarly, AI is accelerating the development of multifunctional nanoprobes and facilitating multimodal image fusion and cross‐scale analysis, further enhancing imaging performance and supporting comprehensive, high‐precision characterization of retinoblastoma and its boundaries.

Despite these advances, several critical barriers continue to impede clinical translation of nanoprobes for retinoblastoma imaging. First, tumor lesions and their peripheral margins are highly heterogeneous, with substantial interpatient and intertumoral variability in target expression. This variability compromises targeting consistency and weakens boundary discrimination. Second, multimodal imaging still faces challenges in spatial registration, complementary information integration, and hierarchical feature fusion, limiting the transition from qualitative boundary description to quantitative assessment. Third, currently available animal models differ markedly from humans in ocular anatomy, BRB permeability, and immune microenvironment, thereby reducing the predictive value of nanoprobe biodistribution and therapeutic response. Fourth, the intraocular metabolism, clearance pathways, immunogenicity, and potential retinal toxicity of nanoprobes remain insufficiently understood, raising important biosafety concerns.

To address these challenges, future research should focus on several key directions. (1) Greater emphasis should be placed on target discovery and multi‐target synergistic strategies, with integration of tumor microenvironment‐responsive elements and multi‐physical‐field‐driven mechanisms to improve coverage of tumor cores, infiltrative margins, and disseminated foci. (2) AI‐driven multimodal image fusion should be further developed to enable quantitative delineation of structural and functional tumor boundaries, ideally supported by multicenter data sets to establish standardized evaluation frameworks. (3) Human‑derived retinoblastoma organoid models, intraocular pharmacokinetic modeling, and BRB transport simulations should be incorporated to better bridge the gap between interspecies differences and enhance translational feasibility from preclinical studies to clinical application. (4) Future probe design should prioritize biodegradable and metabolizable nanosystems with well‐defined clearance pathways, together with systematic long‐term evaluation of ocular biosafety. (5) Integration of multimodal imaging, intelligent nanoprobes, and AI‐assisted image analysis has the potential to provide objective boundary measurements that support individualized treatment selection, intraoperative guidance, therapeutic monitoring, and recurrence surveillance. Such developments may ultimately facilitate the translation of advanced imaging technologies from laboratory research to routine clinical management of retinoblastoma.

Importantly, although many of the nanoprobe engineering strategies, multimodal imaging technologies, and AI‐assisted analytical methodologies discussed in this review are broadly applicable across oncology, their implementation in retinoblastoma requires careful adaptation to the unique biological and clinical characteristics of this disease. Unlike most solid tumors, retinoblastoma develops within the confined intraocular environment, where preservation of ocular structures and visual function is of paramount importance. Moreover, the presence of the BRB, frequent vitreous seeding, the pediatric patient population, and the need for ultra‐high‐resolution boundary delineation impose distinct challenges for nanoprobe delivery, imaging sensitivity, and clinical translation. Therefore, future research should prioritize the development of retinoblastoma‐tailored imaging strategies that integrate disease‐specific targeting, multimodal imaging, and AI‐assisted quantitative analysis, rather than directly extrapolating technologies established for other cancer types.

In summary, the integration of materials science, ophthalmology, imaging technology, and computational science is expected to substantially advance early risk identification, screening, and quantitative boundary delineation in retinoblastoma. Nanoprobe‐assisted multimodal imaging technology is reshaping the diagnostic and therapeutic paradigm of retinoblastoma, moving beyond conventional structural imaging toward comprehensive visualization of tumors together with their structural and functional boundaries. Such progress holds considerable promise for improving survival outcomes while maximizing visual preservation in affected patients.

## Author Contributions

**Xuefei He:** writing – original draft, conceptualization. **Precious Amarachi Ikwuagwu:** writing – original draft, writing – review and editing. **Wenjing Pang:** writing – original draft. **Aomen Chen:** writing – original draft. **Lingchao Xiang:** writing – original draft. **Feng Chen:** conceptualization. **Chen Xu:** writing – review and editing, funding acquisition, supervision, validation. **Aiguo Wu:** funding acquisition, supervision, validation.

## Ethics Statement

The authors have nothing to report.

## Consent

All participants provided informed consent.

## Conflicts of Interest

The authors declare no conflicts of interest.

## Data Availability

The authors have nothing to report.
